# Ego-depletion and motor skill performance under pressure—experimental effects of a short term virtual-reality based mindfulness breathing meditation with integrated biofeedback

**DOI:** 10.1038/s41598-024-68043-0

**Published:** 2024-07-30

**Authors:** Matthias Wagner, Alissa Wieczorek

**Affiliations:** Faculty of Human Sciences, Institute of Sport Science, University of the Bundeswehr Munich, Werner-Heisenberg-Weg 39, 85577 Neubiberg, Germany

**Keywords:** Self-control, Sports performance, Breathing-meditation, Biofeedback virtual reality, Stroop-effect, Psychology, Human behaviour

## Abstract

Ego-depletion describes a state of mind, where the capacity for self-control is temporarily depleted after a primary self-control action. The aim of this study was to investigate whether a brief virtual reality-based mindfulness breathing meditation with integrated biofeedback can be considered an effective strategy to counteract the detrimental effects of ego depletion on motor skill performance under pressure. The study included two experiments, each of them designed as counterbalanced cross-over trials and based on an a priori sample-size calculation. Within each experiment, participants completed two appointments in a randomly assigned order, during which they were asked to perform 20 basketball free throws (*N* = 18; Experiment 1) or 20 penalty kicks at a football goal in four target squares (*N* = 16; Experiment 2) under pressure pre and post the following conditions: Stroop-test-induced ego depletion followed by a 15 min resting break, Stroop-test-induced ego depletion followed by a 15 min virtual reality-based mindfulness breathing meditation with integrated biofeedback. Results indicate that, in comparison to a resting break, a brief virtual reality-based mindfulness meditation with integrated biofeedback can counteract the detrimental effects of ego-depletion (Experiment 2) and enhance motor skill performance under pressure (Experiment 1, 2) Implications for researchers and practitioners are derived in light of the identified methodological limitations.

## Introduction

“Oh, it’s saved!” Let us go back to the sporting year of 1996. We’re in the semi-finals of the European Football Championship at London's venerable Wembley Stadium, the birthplace of modern football. England versus Germany, penalty shootout. England’s number 6 runs up to the last penalty for the hosts and misses. Shortly after, England is eliminated from their own tournament, Germany goes into the final and wins it by scoring the first golden goal in football history.

When looking at this introductory example from a psychological perspective, it stands to reason, that athletes sometimes cannot withstand the emotional burden in high pressure competitive situations and thus, respond with an erroneous or degraded performance. In pertinent literature, corresponding phenomenon is referred to as choking under pressure. Following Mesagno and Hill^1^, choking under pressure can be understood as an acute and significant drop in skill execution and performance triggered by increased anxiety. Anxiety, in turn, is commonly understood as “an emotion characterized by apprehension and somatic symptoms of tension in which an individual anticipates impending danger, catastrophe, or misfortune.”^2^. With regard to performance losses, Wang et al.^3^ established the basal link between trait anxiety and choking under pressure, while Benny and Banks^4^ provide evidence for the situation-specific significance of state anxiety with regard to the probability of choking. Oudejans et al.^5^ assumed that the impact of pressure-induced anxiety on performance can be explained by athletes’ attention regulation processes. Hereby the authors basically focus on two explanatory approaches, namely distraction- and self-focus-models. Distraction models imply that athletes are more easily distracted by task-irrelevant information when their anxiety increases, leading to shifts in attention away from task-relevant information. It is hereby assumed that, when the processing of respective task-irrelevant information such as worries exceeds a certain level of attentional capacity, the potential for the retrieval of an optimal performance is reduced. Self-Focus models on the other hand suggest that choking occurs because attention is focused on the execution of the movement—either in terms of explicit-monitoring or conscious processing—and thus interferes with its automatic execution when anxiety increases. On the basis of retrospective verbal reports as well as statements generated by concept mapping, the authors conclude that chocking under pressure is rather explained through distraction (i. e. through attention on performance worries) than through self-focus (i. e. through attention to skill execution) theories. Thus, when maximum performance under pressure is required, the ability to volitionally downregulate emotions and control attention seems crucial. The basic possibility of counteracting an anxiety-induced drop in performance through additional effort is anchored in the Attentional Control Theory^6^. However, following Englert and Bertrams^7^, by hands of respective theory it remains unclear “which self-regulatory processes determine whether or not anxious individuals can counteract detrimental anxiety effects.”. Corresponding regulatory processes complementing the executive functions in restraining impulses and resisting temptations^8^ were summarized by Baumeister et al.^9^ under the collective term self-control. Within their eponymous strength model of self-control, Baumeister et al.^9^ assume that the volitional act of thought-, emotion- and attention-related self-control is energized by a central metaphorical resource. From this perspective, performance differences under pressure are due to trait and subsequent state related differences in that particular resource. If we come back to our introductory example, then we should actually assume that professional athletes are repeatedly exposed to these high-pressure situations and consequently, that their capacity for self-control is adapted on a corresponding high and stable level. However, if this is the case, why did England’s number 6, who at the time already played more than 150 professional matches, ultimately fail? To that extent, Baumeister et al.^9^—similar to Marcora’s et al.^10^ concept of mental fatigueness—offer the hypothesis, that the capacity for self-control can be temporarily depleted after a previous (primary) self-control action, a state called ego-depletion. Corresponding self-control actions leading to ego-depletion for example could imply a regulation of state anxiety, not thinking about a possible failure or fading out irrelevant stimuli in competitive situations. In line with Baumeister’s hypothesis, Englert^11^ found a substantial number of primary research studies which provide evidence for the depletability of state-related self-control strength and subsequent negative impact on sporting performance under pressure. The study by Dorris et al.^12^ serves as an example to illustrate this effect in the following. The authors investigated whether ego depletion can reduce athletes’ persistence at a routine physical exercise by hands of two within-subject experiments. Competitive rowers attempted to complete as many press-ups and competitive hockey and rugby players attempted to complete as many sit-ups as possible after completing an easy and a difficult cognitive task, respectively. Within both experiments, competitive athletes completed fewer repetitions in the difficult task phase in comparison to the easy task phase. Following Dorris et al.^12^, “These findings indicate that athletes’ exercise routines are susceptible to ego depletion and that the strength model of self-regulation is applicable to athletic performance.”. Building up on respective research, Brown et al.^13^ conclude that the effects of prior cognitive exertion on physical performance may even have been underestimated in previous meta-analysis. On the flip side, it should not go unmentioned that the (external) validity of the strength model of self-control^14^, the amount of the ego-depletion effect^15^ or even the replication of corresponding effect^16^ is also viewed critically within parts of the scientific community. Focusing on motivational, emotional and attentional shifts following a first self-control task as discussed by Inzlicht and Schmeichel^17^ instead, is considered a promising alternative to the assumption of a depleted resource. However, we nevertheless have to state that corresponding alternative hypothesis as displayed within the collection by Englert, Graham and Bray^18^ are still lacking sufficient replicative evidence and thus, consider it reasonable to proceed with the basic assumption offered by Baumeister et al.^9^. It can now be assumed that professional athletes will not have to struggle with fear of failure for the entire duration of the game; if this particular capacity for self-control was thus presumably not depleted significantly until the penalty shoot-out, why was England’s number 6 nevertheless unable to withstand the emotional burden in that decisive moment? To that extent it is important to notice, that the effect of ego-depletion is assumed to have a cross-domain character, indicating for example that a primary attention regulation task can impact on the ability to regulate one’s emotions during a subsequent (e. g. motor skill,^19^) task. In other words, if we assume that having already played 120 min plus on the highest level obviously required numerous and various actions of self-control, this resource-depleting cognitive preload presumably made it impossible to downregulate upcoming emotions and successfully manage this high-pressure “The whole of England is with you”-situation. Thus, the primary question of interest from the perspective of applied performance psychology is after effective measures to restore the depleted self-control resource in order to enable athletes to deliver their individual peak performance when demanded.

The concept of mindfulness has received increasing attention in research and public media over the past 20 years^20^. In order to specify empirically falsifiable theoretical predictions for the purpose of validation and refinement, Bishop et al.^21^ proposed an operational definition which covers today’s general understanding of the concept. Hereafter, mindfulness is described “[…] as a kind of non-elaborative, non-judgmental, present-centered awareness in which each thought, feeling, or sensation that arises in the attentional field is acknowledged and accepted as it is.” (p. 232)^21^. Mindfulness has been introduced to the field of psychology, especially with respect to the clinical context, by evaluated therapeutic approaches such as the Mindfulness-Based Stress Reduction (MBSR)^22^ program or the Acceptance and Commitment Therapy (ACT)^23^. Both mindfulness-based interventions (MBIs) have originally been conceptualized for patients in pain, with depression or social anxiety but are constantly evolved for other target groups.

In (professional) sports, mindfulness has gained increasing attention by prominent multipliers such as legendary basketball coach Phil Jackson. One of the most prominent MBIs in sports is the Mindfulness-Acceptance-Commitment (MAC) approach^24,25^. With reference to MBSR, and ACT, MAC integrates fundamental conceptual elements of mindfulness like a present-focus awareness and attention, a non-judgmental and acceptance approach to situations, physical sensations and emotions as well as an openness and curiosity toward experience and last, compassion for self and others^26^. Heinz et al.^27^ illustrate the use of MAC by the example of a tennis player with competitive anxiety as follows. By reflecting on the own experiences, the tennis player's awareness for the connection between performance failure and dysfunctional emotions and cognitions is build up first. Following an increased awareness, a mindful attitude in sports situations as well as in everyday life contexts is developed and practiced via various mindfulness exercises such as breathing mediation. In addition to the mindfulness exercises, acceptance-promoting techniques are practiced; by doing so, the tennis player should learn to no longer perceive thoughts as a reality that guides action in order to ultimately relativize the significance of (negative) thoughts for their own performance. In the further course of the program, special emphasis is set on developing the tennis player's life and performance-related values in order to recognize emotion-driven behaviour and to reinforce opposing, value-driven behaviour. The final stage of the program is about stabilizing and habitualizing the newly learned behaviours and ways of thinking. For a broader range of MBIs including MAC, most current systematic review and meta-analysis by Wang et al.^28^ “[…] provided preliminary support for the effectiveness of MBIs in promoting athletic performance, mindfulness level, and mindfulness-related psychological components among athletes.” (p. 13). Psychological components affected by corresponding mindfulness interventions were acceptance, flow, psychological flexibility as well as ruminative response and the pooled effect size was calculated with *d* = 0.81. Following Birrer et al.^29^, corresponding effects can be explained through various impact mechanism including the enhancement of the athlete`s ability to downregulate negative emotions by accepting those intrusive dysfunctional thoughts instead of trying to actively suppress them. In other words, both formal and informal mindfulness practices could obviously be supportive in preventing constant rumination which in turn has a positive influence on attentional and motor control skills.

Over and beyond, under the condition of a regulative cognitive preload and resulting low resources, Friese et al.^30^ evidenced the perspective, that “[…] a brief period of mindfulness meditation may serve as a quick and efficient strategy to foster self-control […]” (p. 1016). In other words, mindfulness practice not only seems beneficial for developing the ability to self-control in the long run, but also in terms of a brief impulse to restore corresponding resource in case of depletion.

The interface between ego-depletion, mindfulness and sporting performance was initially investigated by Stocker et al.^31^. The authors could not provide evidence for the assumed compensating effect of a brief four-minute mindfulness exercise on basketball free-throw performance in a state with temporarily depleted self-control strength. With reference to Englert and Bertrams^32^, Stocker et al.^31^ discuss a potential lack of intervention-induced relaxation as one shortcoming of their study which on the other hand puts emphasize on the importance of that particular aspect in selecting appropriate interventions in future studies. Subsequent research was provided by Shaabani et al.^33^. The authors also aimed to examine whether a brief (15 min) breath and body mindfulness intervention can counteract the detrimental effects of ego-depletion in basketball free throw under pressure. Therefore, Shaabani et al.^33^ investigated 72 experienced male basketball players between 20 and 35 years of age. Those individuals were randomly assigned to four different groups, whereas the comparison between the depletion/mindfulness- and the depletion/no mindfulness-group is of particular interest for our context. In both groups, participants started with 30 basketball free throws at baseline during which they listened to distracting audio messages via stereo headphones to induce performance worries. Competitive pressure was induced by informing participants that their individual and team performance would be ranked and made public among participants. A modified Stroop color-word test was used to manipulate self-control and to induce the state of ego-depletion in the following. The Stroop-test—one of the most frequently administered self-control tasks is based on the two-task-paradigm, which implies that a manipulation of situational self-control strength (incongruent condition) in comparison to a control (congruent) condition in a primary task leads to an exhaustion of this particular resource, detectable in a secondary task requiring self-control^34^. Right after the manipulation and depending on their group assignment, participants either received a fifteen-minute audio-based mindfulness intervention with focus on breathing patterns and physical body sensations or a fifteen-minute audio-based history lesson. In both groups, experiment ended with 30 posttest free throws. When contrasting pre- and posttest performance, shooting score decreased within the depletion/no mindfulness-group and remained rather stable within the depletion/mindfulness-group. Following their results and in accordance with the non-sport related findings by Friese et al.^30^, the authors conclude “[…] that a brief mindfulness intervention may effectively mitigate the influence of ego depletion on the performance of these perceptual motor skill tasks.” (p. 209p). What has to be pointed out here is, that Shaabani et al.^33^ used a between-subject design, which leaves open the possibility, that even though participants in both groups reported a similar amount of subjective depletion in the immediate manipulation check, the objective impact of the depletion on the immediate post performance could have been different. In other words, it is not ultimately clear whether the detrimental effect of ego-depletion shown in the depletion/no mindfulness-group displayed comparably substantial in the depletion/mindfulness-group or was present here at all; this latter situation in turn would explain the higher performance alternative to the assumed compensatory effect simply as a result of a non-manipulation response in the depletion/mindfulness-group. Strictly speaking, the only thing we can actually derive from existing data is, that performance in the depletion group is diminished after a ten-minute audio-break and that performance in the depletion/mindfulness-group comes close to the group-specific baseline-performance after a time-equivalent mindfulness intervention; corresponding data-interpretation gives rise for the idea of replicating the primary study using a within-subject design.

Virtual reality- (VR) based technologies are increasingly attracting the attention of users and researchers since they promise to set a milestone in technological innovation^35^. Following the current definitional approach by Rauschnabel et al.^36^ “Virtual Reality is an artificial, virtual, and viewer-centered experience in which the user is enclosed in an all-encompassing 3D space that is—at least visually—sealed off from the physical environment.”. The all-encompassing user-experience mainly depends on the degree of immersion, describing the applications potential to convey a sense of presence. Actually being part of the virtual world can be fostered through a multisensory integration of as many perceptual senses as possible as well as through the accuracy, resolution and reactivity in the production of the stimuli^37^. In simulating a realistic world that responds to real-time interaction and evokes a sense of presence, VR can facilitate the visualization and reification of abstract content into audiovisual stimuli to enable the transformation of information in ways that are limited in physical environment^38^. That makes VR an exceptional medium for gaming and entertainment, but more important in our context a medium to positively influence for example emotional well-being^39^. Combined with biofeedback, VR offers many benefits as the virtual environment is fully controllable and customizable and therefore offers a safe and supportive training field. Unwanted external audiovisual stimuli can be blocked and replaced by desired and task-relevant stimuli reducing distraction and supporting a sustained focus on the task^40^. VR scenarios are nowadays mostly realized via head-mounted displays (HMDs) and show a wide range of applications from business via healthcare to research (in overview^36^).

Existing empirical evidence on the application of interactive VR to sport has been addressed in a recent systematic review by Neumann et al.^41^. The authors conclude that VR-based systems seem promising in terms of an adjunct to real world training. However, they also critically note that "[…] most research has been conducted on endurance sports such as running, cycling, and rowing, and further research is needed to investigate the use of interactive VR in skill-based sports. (p. 183)". In this respect, a more recent review by Farley et al.^42^ suggests a growing body of evidence.

By shielding the outside world and creating an interesting and immersive environment, VR naturally provides a suitable setting for mindfulness training^43^. However, research on the potential of VR to improve the effectiveness of MBIs displays mixed evidence. Within their systematic review Zhang et al.^44^ conclude that MBI retained more participants under the VR-condition and that VR-based MBI led to significant reductions in anxiety, stress, depression, mindfulness, emotional state, and pain level; however, in light of the conflicting findings that become apparent when comparing the primary studies by Gromala et al.^45^, Navarro-Haro et al.^46^ and Wayment et al.^47^ it remains unclear, whether the VR-environment actually has an additional impact beyond the effects of traditional MBIs. On the flip side, Arpaia et al.^48^, who narratively reviewed the field with special focus set on mindfulness mechanisms like decentering and interoceptive awareness^49^, summarize their findings to the effect that VR-conditions in particular guarantee increasing relaxation, self-efficacy, reduce mind wandering and preserve attention resources.

The interface between mindfulness, virtual reality and sporting performance has not been empirically researched so far to our best knowledge. In the present study, we address this missing link from a sport psychology perspective. With reference to our initial theoretical framework, we aim to identify effective strategies to counteract the detrimental effects of ego depletion on motor skill performance and hereby hypothesize, that a VR-based MBI with integrated biofeedback could be beneficial for this purpose. From a critical rationalist perspective, we are aiming to replicate the primary findings on ego-depletion, mindfulness and sporting performance by Shaabani et al.^33^ under the perspective of a VR-based MBI with integrated biofeedback and in the context of various motor skills as proposed by Neumann et al.^41^. In aiming to prevent miss- or overinterpretation of results due to interindividual differences, we consider a within subject approach an adequate strategy for our intended replication.

## Methods

### Study design and procedure

**Figure 1:**
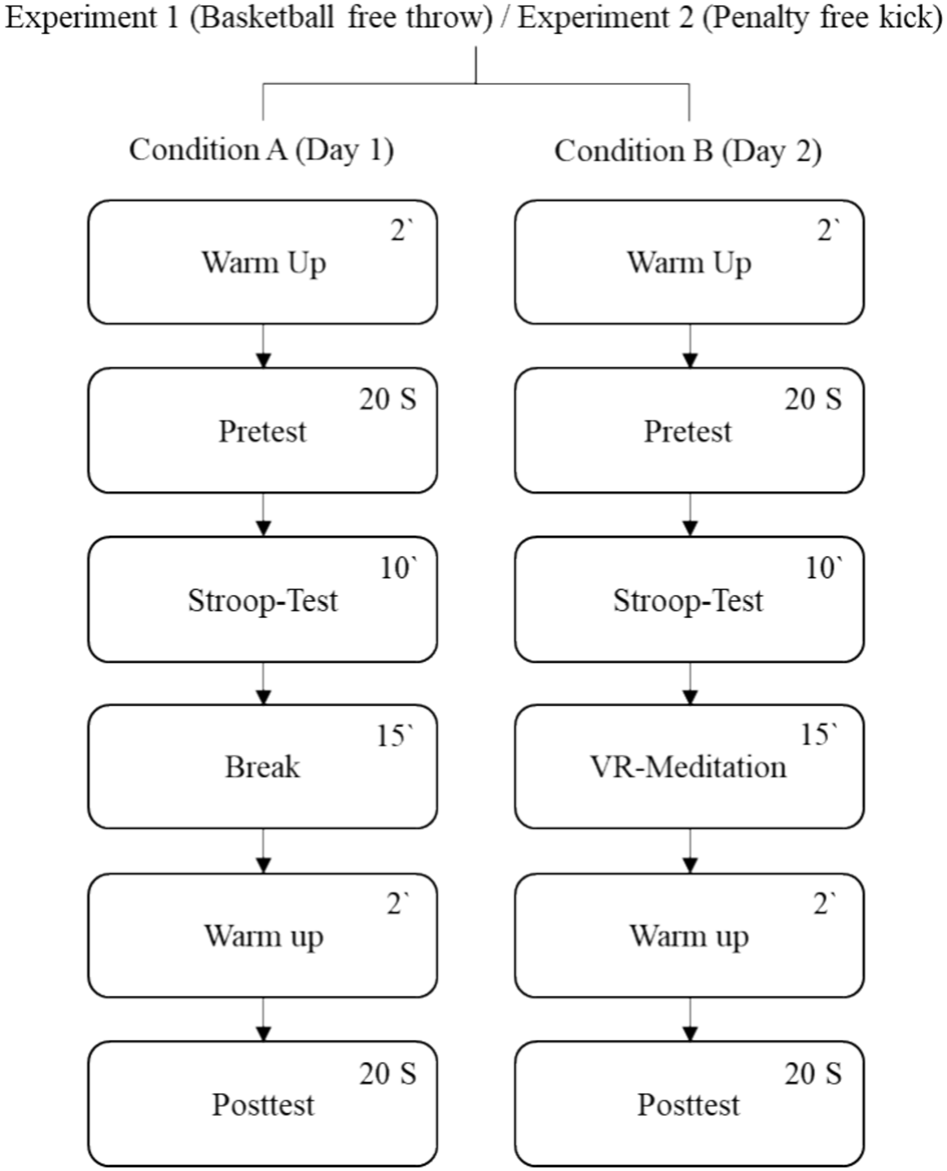
Experimental study design. S = Shots on target under pressure.

The study included two experiments, each of them designed as a cross-over trial and carried out in the sport psychology laboratory at the authors institution. Within each experiment, participants completed two appointments (washout period: 7–10 days) in a randomly assigned order (counterbalanced design; simple randomization), during which they were asked to perform 20 basketball free throws (Experiment 1) or 20 penalty kicks at a football goal in four target squares (predetermined order clockwise; no goalkeeper; Experiment 2) under pressure before (pre) and after (post) the following conditions: 10-min Stroop-test followed by a 15-min resting break (Condition A), 10-min Stroop-test followed by a 15-min VR-based mindfulness breathing meditation with integrated biofeedback (VRMMB; Condition B). Resting break/VRMMB and pretest/posttest/manipulation were each conducted by different members of the research staff. Staff members responsible for testing and manipulation were blinded concerning the condition the test subjects were undergoing. Figure 1 provides a comprehensive graphical representation of the study process in Experiment 1 and 2.

### A priori sample size calculation and recruitment

Within their original study, Shaabani et al.^33^ report a significant difference in the basketball shooting posttest performance between the depletion/no mindfulness- (*N* = 18; mean = 40.73%, *SD* = 8.72%) and the depletion/mindfulness—(*N* = 18; *mean* = 49.39%, *SD* = 8.32%) group. Thus, when aiming to replicate corresponding effect (*d* = 1.02) two-tailed with a priori alpha = 0.05 and a desired power of 1−*ß* = 0.80, 17 participants would represent an optimal sample size. Participants for both experiments were recruited via internal mailing lists in the authors institutional context. Prerequisite for participation in one out of the two respective experiments were the presence of previous basketball (Experiment 1) or football (Experiment 2) experiences, respectively defined as regular free time play, previous playing in the context of school sports, or actively playing in a club.

### Experimental set-up and outcome measures

The experimental basketball set up (Experiment 1) included a game ball (size 7; weight: 1.4 pounds), a basketball hoop (ring height: 10 feet; diameter: 18-inch inside) and a free throw line (distance to the hoop: 15 feet). The experimental football set up (Experiment 2) included a game ball (size L, 0.99 pounds), a football goal (width: 9.84 feet, height: 6.56 feet, depth: 3.35 feet, weight: 35.27 pounds) with four same sized (16.15 square feet) target squares and a penalty point (distance to goal: 22.97 feet). Black and yellow routing tape was used to mark the free throw line (Experiment 1) or the penalty point (Experiment 2) on the floor, respectively and to fix the target squares (Experiment 2) between posts and crossbar of the football goal. The experimenter moved laterally between the free-throw line and the basketball hoop or the penalty point and the goal, respectively to return the game ball. During pre-and post-testing, subjects wore a wireless headset connected to the experimenter's laptop (placed lateral to the throwing/shooting scenario), through which they listened to twenty-one distracting sentences (e. g.: “It’s going badly, I really need to improve now”, “How is it possible that I made a mistake?”) to induce performance worries. Respective sentences were translated from Nieuwenhuys and Oudejans^50^ and spoken by a male and a female voice as unemotionally and monotonously as possible. To induce pressure, participants were explicitly informed about their trial being video-taped with reference to Wolch et al.^51^.

### Experimental manipulation

**Figure 2:**
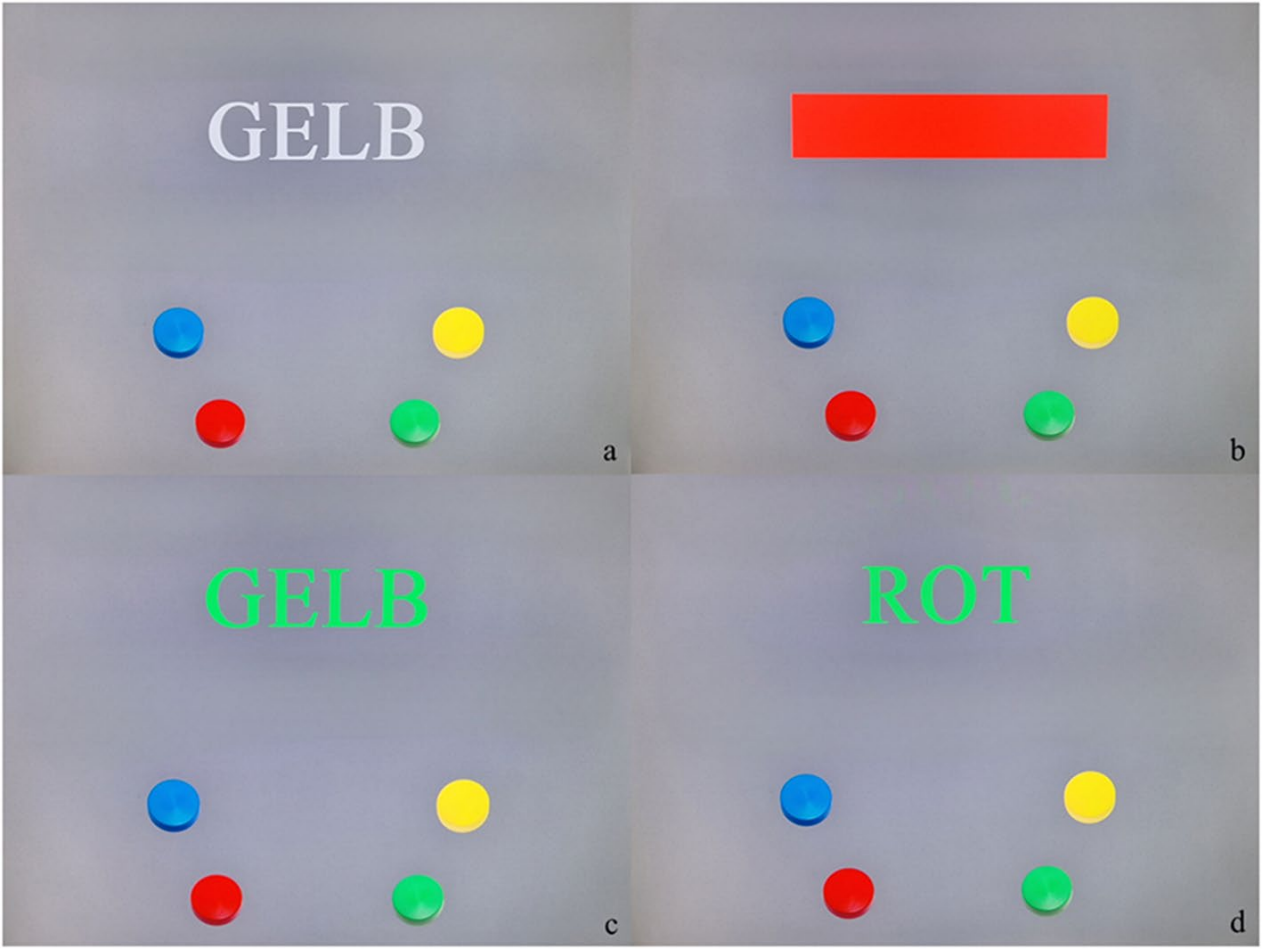
Stroop Interference Test (Version 51)^52^.

The German version of the Stroop Interference Test (SIT)^52^ was used to experimentally manipulate self-control strength and to evoke the state of ego depletion in both experiments. SIT represents a sensorimotor speed test that measures speed performance in reading words and naming colors as well as under color-word interference condition allowing for an evaluation of individuals information processing and attention processes. To ensure the first task is of sufficient duration to reliably induce ego-depletion^53^, we chose SIT testform S7 which requires an average of approximately 10 min to complete. Within S7, color words without coloring (Fig. 2a; 128 trials) or simple color bars are given (Fig. 2b; 128 trials) at baseline. The color words are then presented in different antagonistic colors. Here, depending on the task, the test person reacts exclusively to the font color (Fig. 2c; 128 trials) or the meaning of the color word (Fig. 2d; 128 trials). In every task, the test person presses a color key on the panel or touchscreen as quickly as possible^52^. SIT has been shown to provide objective, reliable and valid data^52^ on the intended Stroop-effect^54^. Successful applications of various SIT-modifications in sport psychology research displaying a stroop-induced state of ego-depletion and a subsequent performance decrease can be found in the primary study of interest^33^ as well as in a number of prior original studies^55,56^.

To ensure successful inducement of the ego-depletion effect, we administered the manipulation-check scale used by Shaabani et al.^33^ immediately after manipulation under both conditions. The scaled comprised four items (“How difficult did you find the task?’’, “How effortful did you find the task?”, “How mentally depleted do you feel at the moment?”, “When reporting the color of the words, how difficult was it to suppress the meaning of the words?) which had to be answered on a 7-point Likert-type scale from 1 (not at all) to 7 (very much) and displayed a good internal consistency (Cronbach’s α = 0.80).

### Experimental treatment

**Figure 3:**
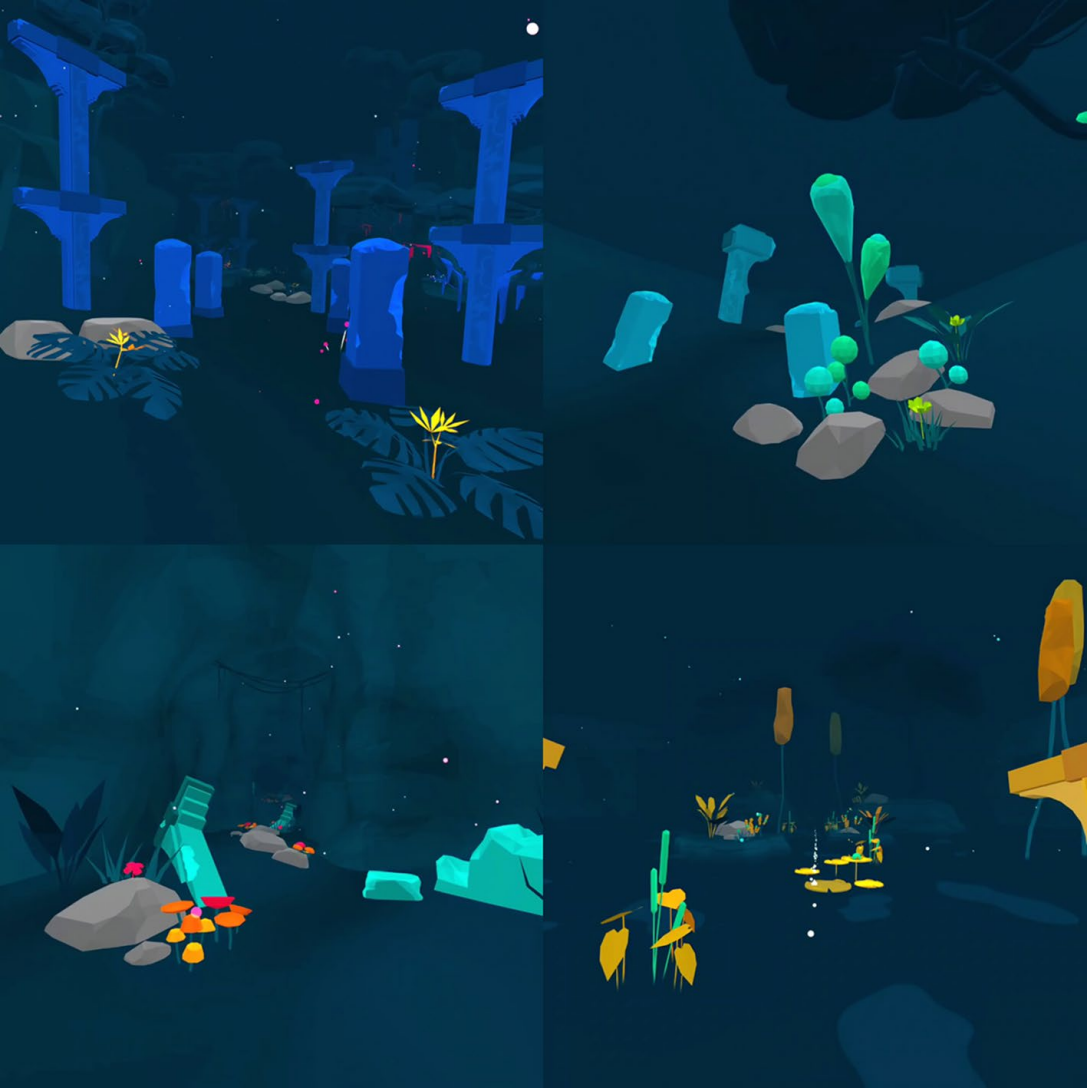
Virtual reality-based mindfulness breathing meditation with integrated biofeedback (Flowborne, Version 1.50)^58^.

To counteract the induced ego-depletion effect, participants conducted a 15-min VRMMB termed Flowborne^57^. Flowborne is a meditative breathing game with biofeedback support created by psychologists to foster an intuitive way of learning a calming diaphragmatic breathing style. To support respective learning process, the VR controller detects the subtle respiration-induced movements of the gamer’s abdomen. Resulting breathing information induces real-time adaptations of the virtual environment in terms of every exhale being translated into a floating movement which guides the gamer’s attention on their breath and in the following, helps to improve their breathing style through a highly individualized approach. Corresponding information were provided to all participants before starting their test series under condition B. Flowborne runs on Meta Quest 2, a head-mounted display which is considered a state-of-the-art mobile VR system since it allows participants to deeply immerse themselves in a nonobtrusive way^58^. In terms of empirical evidence of the Meta Quest 2-based Flowborne meditation (see Fig. 3), Rockstroh et al.^59^ showed, that six eight-minute Flowborne-sessions resulted in a number of positive outcomes such as increased perceived breath awareness, improved diaphragmatic breathing, increased relaxation, decreased perceived stress, reduced symptoms of burnout and boosted relaxation-related self-efficacy. The decision for the 15-min meditation interval—as well as the corresponding resting break—was due to the replicative nature of our study. Shaabani et al.^33^ hereby refer to Arch and Craske^60^, who ultimately legitimize their approach through the recommendations provided by Kabat-Zinn^22^ and Segal^61^.

### Data analysis

We used the video recordings to ensure the objectivity of our scoring in both experiments. Assumption of normality was tested for pre- and posttest data under Condition A and B using Shapiro Wilk tests (SWT)^62^ as justified within the power-comparison study by Razali et al.^63^. Two one-sided tests (TOST)^64^ were conducted to test for equivalence of the baseline performances under both conditions. In case of non-equivalence, baseline difference score was calculated and integrated as covariate in subsequent analysis. Pre-and posttest data were structured with respect to the within-subject design and analyzed using a two-way Analysis of Variance (ANOVA) with repeated measures in both experiments. In case of a significant interaction between both conditions, overall analysis was followed by two separated one-way ANOVA with repeated measures to interpret corresponding main effects. Significance level for all statistical tests was set a priori to α = 0.05 (two-tailed) to control for type I error. Effect sizes (part. *η*^*2*^) and post-hoc power (1−*ß*) were calculated to specify manipulation and treatment effects and to justify the decision for the alternative hypothesis in case of significance. All data were analyzed using IBM SPSS Version 29^65^.

### Ethics statement

This study was reviewed and approved by the ethical commission of the University of the Bundeswehr Munich. Participants provided their written informed consent to participate in this study. The authors confirm that all research was performed in accordance with relevant guidelines/regulations.

## Results

### Experiment 1

#### Sample description and assumption of normality

Experiment 1 included 18 (61.1% male) participants with a mean age of 23.89 years (*SD* = $\pm$ 4.14 years). No violation of the assumption of normality concerning the free-throw performance distributions could be detected at any measurement point (Pretest Condition A: *W*(18) = 0.95, *p* = 0.455; Posttest Condition A: *W*(18) = 0.95, *p* = 0.379; Pretest Condition B: *W*(18) = 0.95, *p* = 0.498; Posttest Condition B: *W*(16) = 0.94, *p* = 0.261) allowing for a parametric data-analysis (Table 1).

**Table 1:** Descriptive results.

		N	Age	Gender	Pretest score	Posttest score
Experiment 1	Condition A	18	23.89 (3.14)	Male (61.1%)	5.56 (27.8%) (2.26)	6.72 (33.6%) (2.27)
	Condition B				5.78 (28.9%) (1.77)	7.78 (38.9%) (3.37)
Experiment 2	Condition A	16	23.06 (2.44)	Male (100%)	16.44 (82.0%) (1.46)	15.19 (76.0%) (2.29)
	Condition B				15.50 (77.5%) (2.73)	17.81 (89.1%) (1.52)

#### Manipulation check

Participants scored higher on the manipulation check scale under Condition A (mean sum score = 13.83 points, SD =  ± 3.99 points) and B (mean sum score = 15.72 points, SD =  ± 4.25 points) in comparison to the primary study by Shaabani et al. (^33^; Depletion group: mean sum score = 9.74 points, SD =  ± 2.21 points; Depletion/Mindfulness group: mean sum score = 9.43 points, SD =  ± 1.83 points) indicating a subjectively stronger depletion.

#### Depletion and meditation effects on performance

Baseline scores under Condition A (*mean* = 5.56 points, *SD* = $\pm$ 2.25 points) and B (*mean* = 5.78 points, *SD* = $\pm$ 1.77 points) displayed no equivalence (TOST Upper:* t* (32.2) = 0.41, *p* = 0.342; TOST Lower: *t* (32.2) = − 1.07, *p* = 0.146) necessitating the integration of respective difference score as covariate. Two-way ANOVA with repeated measures indicated no significant interaction between Condition A and B (*F* (1, 17) = 1.05, *p* = 0.321, part. η^2^ = 0.061; 1−*ß* = 0.16) allowing for an independent interpretation of respective main effects. Main effects indicated that participants did not significantly differ in their basketball-scoring performance before (Pretest Condition A: *mean* = 5.56, *SD* = $\pm$ 2.25, 27.8%) and after (Posttest Condition A: *mean* = 6.72, *SD* = $\pm$ 2.27, 33.6%) manipulation (*F* (1, 17) = 1.05, *p* = 0.321, part. η^2^ = 0.061; 1−*ß* = 0.16) and that basketball-scoring performance significantly increased when manipulation was followed by mindfulness meditation (Pretest Condition B: *mean* = 5.78, *SD* = $\pm$ 1.77, 28.9%; Posttest Condition B: *mean* = 7.78, *SD* = $\pm$ 3.37, 38.9%; *F* (1, 17) = 11.41, *p* < 0.05, part. η^2^ = 0.416; 1−*ß* = 0.89). In light of the achieved high post-hoc power, the alternative hypothesis can be considered a plausible explanation for the detected large effect under Condition B.

### Experiment 2

#### Sample description and assumption of normality

Experiment 2 included 16 male participants with a mean age of 23.06 years (*SD* = $\pm$ 2.44 years). No violation of the assumption of normality concerning the penalty-kick performance distributions could be detected at any measurement point (Pretest Condition A: *W* (16) = 0.92, *p* = 0.183; Posttest Condition A: *W* (16) = 0.98, *p* = 0.924; Pretest Condition B: *W* (16) = 0.97, *p* = 0.885; Posttest Condition B: *W* (16) = 0.93, *p* = 0.286) allowing for a parametric data-analysis.

#### Manipulation check

Participants scored higher on the manipulation check scale under Condition A (mean sum score = 15.75 points, SD = $\pm$ 3.32) and B (mean sum score = 15.06 points, SD = $\pm$ 3.45 points) in comparison to the primary study by Shaabani et al. (2019; Depletion group: mean sum score = 9.74 points, SD =  ± 2.21 points; Depletion/Mindfulness group: mean sum score = 9.43 points, SD =  ± 1.83 points) indicating a subjectively stronger depletion.

#### Depletion and meditation effects on performance

Baseline scores under Condition A (*mean* = 16.44 points, *SD* = $\pm$ 1.46 points) and B (*mean* = 15.30 points, *SD* = $\pm$ 2.84 points) displayed no equivalence (TOST Upper:* t* (18.1) = 2.07, *p* = 0.027; TOST Lower: *t* (18.1) = 0.86, *p* = 0.800) necessitating the integration of respective difference score as covariate. Two-way ANOVA with repeated measures indicated a significant interaction between Condition A and B (*F* (1, 15) = 33.89, *p* < 0.001, part. η^2^ = 0.708; 1−*ß* = 1.00) requiring an independent analysis of corresponding main effects within two separate one-way ANOVA with repeated measures. Main effects indicated that participants scored significantly fewer goals following manipulation (Pretest Condition A: *mean* = 16.44, *SD* = $\pm$ 1.46, 82.0%; Posttest Condition A: *mean* = 15.19, *SD* = $\pm$ 2.29, 76.0%; *F* (1, 15) = 9.15, *p* < 0.01, part. η^2^ = 0.379; 1−*ß* = 0.81) and that the number of goals increased when manipulation was followed by mindfulness meditation (Pretest Condition B: *mean* = 15.50, *SD* = $\pm$ 2.73, 77.5%; Posttest Condition B: *mean* = 17.81, *SD* = $\pm$ 1.52, 89.1%; *F* (1, 15) = 36.22, *p* < 0.001, part. η^2^ = 0.707; 1−*ß* = 1.00). In light of the achieved high post-hoc power, the alternative hypothesis can be considered a plausible explanation for the detected large effect under Condition A and B.

## Discussion

The aim of this study was to investigate whether a brief virtual reality-based mindfulness breathing meditation with integrated biofeedback (VRMMB) can be considered an effective strategy to counteract the detrimental effects of ego depletion on motor skill performance under pressure. Therefore, a basketball free-throw scenario (Experiment 1) as well as a penalty-shoot out scenario (Experiment 2) was set up in orientation to the primary study by Shaabani et al.^33^. Both experiments were designed as counterbalanced cross-over trials. Recruitment of participants (Experiment 1: *N* = 18; Experiment 2: *N* = 16) followed an a priori sample size calculation based on the effect size reported within the primary study. Self-control strength was manipulated by a ten-minute Stroop Interference Test (SIT) followed by a fifteen-minute break (Condition A) or a fifteen-minute VRMMB (Condition B), respectively.

Within Experiment 1, participants reported a stronger depletion after the manipulation than in the primary study; however, their basketball free-throw performance under pressure did not significantly differ between pre- and posttest under Condition A. Even though individuals in Experiment 1 obviously did not respond with an expected performance decrease following the successful manipulation, there performance significantly increased from pre- to posttest by 10% under Condition B. Thus, our results obtained from Experiment 1 at least indicate that a fifteen-minute VRMMB could enhance performance in a basketball free-throw scenario under pressure. Corresponding interpretation of results is in line with Shaabani et al.^33^ who conclude “[…] that mindfulness may help athletes improve their performance in perceptual-motor tasks under pressure […]” (p. 209). In light of the fact that performance increase in the primary study (Δ_Post-Pre_ =  + 4.18%) was substantially lower than in the present replication, our results give rise to the assumption of an MBI-enhancing VR effect to be addressed below.

Within Experiment 2, participants again reported a stronger depletion after the manipulation than in the primary study. In opposite to Experiment 1, penalty-shoot out performance significantly decreased from pre- to posttest by 6% under Condition A as expected (primary study: Δ_Post-Pre_ = − 12.5%); on the flip side, performance significantly increased from pre- to posttest by 11.6% under Condition B (primary study: Δ_Post-Pre_ = − 1.7%). Therefore, our results obtained from Experiment 2 indicate that a fifteen-minute VRMMB can not only enhance performance similar to Experiment 1, but also that respective measures can be supportive in counteracting the detrimental effects of ego-depletion in an experimental penalty shoot-out under pressure. The latter results are again in line with Shaabani et al.^33^ wo state “that a brief mindfulness intervention may effectively mitigate the influence of ego-depletion on the performance […]”.

This study provides new insights under three perspectives. First, both experiments were set up in terms of replicating a recent primary study on the potentials of short-term MBI in counteracting the detrimental effects of ego-depletion on motor skill performance. Thus, corresponding results contribute to an iterative extension of this particular body of knowledge. Second, this study sheds light on the potentials of VR in sports beyond the hitherto mainly addressed endurance tasks by focusing different motor skills in line with Craig et al.^66^ as well as Covaci et al. ^67^. Third, this study offers cutting-edge research since both experiments indicate, that the benefits of conducting MBI in a VR-environment^48^ are obviously also given in the so far unexplored sporting context.

How can our results be embedded in and explained out of the existing body of knowledge? If we believe in a substantial impact of the ego-depletion effect on performance as concluded out of Experiment 2 and the preceding original papers reviewed by Englert^11^ as well as our primary source of research, our corresponding results are in line with those evidencing the possibility of counteracting the detrimental effects of ego-depletion on sporting performance^33,68^ and in general^30^ by appropriate interventional measures. Our particular treatment hereby presumably works as follows: The VRMMB Flowborne fosters an increased relaxation^59^ out of which a replenishment of the self’s depleted resources can occur as evidenced by Tyler et al. ^69^ as well as an increased perceived breath awareness^59^ which can reduce the detrimental effects of ego-depletion as evidenced by Alberts et al.^70^. A respective compensation of the cognitive preload presumably enables participants to successfully downregulate their upcoming and—due to pressure, increased—emotions and in consequence, to maintain their performance. The performance enhancement effect observed in Experiment 1 and 2 on the other hand can be explained when considering that MBIs in general are well evidenced to foster the athlete`s ability to downregulate negative emotions^29^, that corresponding effect in turn is evidenced to be particularly pronounced under biofeedback-controlled VR conditions^48^ as with our VRMMB and that our VRMMB is finally evidenced to foster performance enhancing psychological components such as an increased relaxation-related self-efficacy, a decreased perceived stress as well as a boosted relaxation^59^.

The reach of our study results should be reflected in light of certain methodological considerations. First, participants listened to distracting sentences to induce performance worries and were told that their trial was video-taped. However, we did not measure the actual emotional response and thus, can only trust in the effectiveness of respective measures with reference to Nieuwenhuys et al.^50^ and Wolch et al.^51^. Participants emotional response should be addressed in future replication trials via appropriate objective and/or subjective measures to assure an actual necessity for regulation is given in the first place. The same applies to the VRMMB, whose relaxing effect was assumed with reference to Rockstroh et al.^59^ but actually not measured within the present study. Second, we have to keep in mind that under Condition A, manipulation was followed by a 15-min break in both experiments. Following Tyler and Burns^69^, corresponding interval-length might have given participants the time to implicitly recover their resources which presumably explains why we could not observe a performance decrease under Condition A in Experiment 1. However, the pre-post performance difference observed under Condition A in Experiment 2 indicates that the recovery break obviously did not result in a full restorage of self-control strength in that particular set-up; when considering the results observed under Condition B, corresponding results allow to derive a certain compensatory effect of the VRMMB treatment in our view. However, the idea that our comparison Condition A is presumably biased by the recovery-break effect leaves open the possibility, that the true compensatory potential of the VRMMB treatment might even exceed 6%. To unravel the actual potential of our VRMMB treatment, an adequate length of the recovery break (< 10 min; see Tyler and Burns^67^) and consequently, a corresponding adaptation of the treatment length is recommended for future replication trials. Third, we cannot differ whether the observed treatment effects in both experiments are due to MBI or the combination of MBI and VR. Corresponding limitation supports the idea of integrating additional active control groups in an expanded cross-over design for future replication trials; in that sense, our manipulation effect would have been better evidenced in comparison to a non-manipulated control group.

Independently of its origin (MBI, MBI × VR) and actual impact (Experiment 1: performance enhancement; Experiment 2: depletion-compensation. performance enhancement) it has to be stated, that there obviously is a positive treatment-effect on motor skill performance in both experiments. This latter interpretation of results opens the question of their external validity. To that extent, one has to keep in mind that we did not recruit professional athletes but amateur players at best in both experiments. This is important because it is feasible to assume that amateur’s self-control strength is generally lower in such situations due to a lack of exposure to corresponding high-pressure competitive situations. As consequence, amateurs might response more positive to a corresponding short-term intervention than professionals who would potentially not respond at all due to low-threshold. Further, we have to keep in mind that, in comparison to professional players, amateurs tend to benefit from pressure and anxiety^71^ which could also account for our detected treatment effects. Finally, facing the fact that the expertise level of our sample was comparatively low, it cannot be ruled out that the supposed treatment effects simply represent skill-learning effects between pre- and posttest. In sum, a replication with professional players in different sports is indicated in order to foster external validity of our findings from a population-standpoint and thus, to elaborate the true depletion counteracting and performance enhancing potential of our VRMMB.

Professional players are constantly in the focus of attention of spectators on and off screen and thus, without a doubt constantly experience pressure. However, from the perspective of ecological validity, one can certainly question whether the pressure inducement in this study actually comes close to real-life game situations. This issue gives rise for a replication of the present study under field-experiment conditions. When attempting to transfer the laboratory conditions into a corresponding field experiment, two further questions arise: First, it must be questioned whether the SIT manipulation induces a cognitive load comparable to the game-specific regulatory processes and thus represents an adequate correspondence to field conditions. On the other hand, the question arises how the Flowborne VRMMB could be applied in a real-life game situation. To that extent, it is certainly hard to imagine that our penalty kicker from the introductory example enters a shielded virtual reality for a short time before execution, which clearly limits the transferability of our treatment in an ecologically valid scenario.

However, even though ecological validity of our laboratory experiments is naturally limited, corresponding findings nevertheless give rise for an early practical implication as follows. Since we were able to provide evidence for the positive acute effects of our VRMMB on motor skill performance, it is feasible, that an integration of corresponding measures in terms of a formal or informal adjunct to athletes daily or weekly training routines might positively impact on their ability for relaxation and their perceived breath awareness. Building a corresponding basis in turn might enable athletes to counteract the state of ego-depletion and to maintain their performance under pressure even in absence of a supporting VR-HMD. In addition, it is feasible that a regular use of the VRMMB could strengthen the athlete`s ability to downregulate negative emotions as well as their self-efficacy, which in turn can have a positive effect on their performance.

This study provides experimental evidence for the assumption, that, in comparison to a resting break, a brief virtual reality-based mindfulness meditation can counteract the detrimental effects of ego-depletion (Experiment 2) and enhance motor skill performance under pressure (Experiment 1, 2).

## Data Availability

Raw data can be obtained from the authors by request.
